# Validity and reliability of the German versions of the CD-RISC-10 and CD-RISC-2

**DOI:** 10.1007/s12144-021-01670-2

**Published:** 2021-04-09

**Authors:** Anna Irena Wollny, Ingo Jacobs

**Affiliations:** 1 University of Potsdam, Am Neuen Palais 10, Haus 22, 14469 Potsdam, Germany; 2 Medical School Berlin, Berlin, Germany

**Keywords:** Buffer effect, Mental health, Physical health, Trait emotional intelligence, Trait resilience, Psychometric quality

## Abstract

The Connor-Davidson Resilience Scale (CD-RISC) is an established instrument to assess trait resilience. The present study investigates the psychometric properties of the brief German CD-RISC-10 and CD-RISC-2 in an online sample of 360 students. The CD-RISC-10 showed good reliability, whereas the CD-RISC-2 just missed an acceptable level of reliability. The unifactorial structure of the CD-RISC-10 was supported in a confirmatory factor analysis. Correlational analysis with various clinical and non-clinical constructs (e.g., trait resilience, trait emotional intelligence, life satisfaction, well-being, perceived stress, sleep problems, anxiety, depression, and mental and physical health related quality of life) provided evidence for convergent, divergent, and incremental validity of both brief versions of the CD-RISC. Measured with the CD-RISC-10, trait resilience buffered the effects of perceived stress on life satisfaction and aggregated mental health problems, but not on physical health related quality of life. For the CD-RISC-2, a buffer effect was only found for life satisfaction. Taken together, the results of the present study provide support for the validity of the CD-RISC-10 and, to a lesser extent, of the CD-RISC-2. Implications and limitations of the results will be discussed.

## Introduction

Following exposure to adversity, some people are able to recover quickly from stress, to adjust well, and maintain good mental health, or to rise above to overcome adversity. This ability is commonly referred to as psychological resilience (e.g., Aburn et al. 2016). During the last decades, resilience has increasingly become a focus of research in psychological and medical science (Chmitorz et al. 2018; Reich et al. 2010). However, there is no consensual definition of resilience (Aburn et al. 2016). For example, resilience can be defined as

“… *the process of effectively negotiating, adapting to, or managing significant sources of stress or trauma. Assets and resources within the individual, their life and environment facilitate this capacity for adaptation and ‘bouncing back’ in the face of adversity. Across the life course, the experience of resilience will vary*” (Windle 2011, p. 163).

Resilience is thus a complex construct. One established perspective on resilience focuses on personality characteristics (i.e., *trait resilience*) that moderate the adverse effects of stress and promote recovery and adaptation (e.g., Connor and Davidson 2003; Hu et al. 2015). The ubiquitous nature of adversity and its effects on health are likely to stimulate an ongoing interest in resilience, which is accompanied by the urgent need for reliable and well-validated measures of resilience (e.g., Salisu and Hashim 2017; Windle et al. 2011).

One well-known measure of trait resilience is the Connor-Davidson Resilience Scale (*CD-RISC*; Connor and Davidson 2003). The CD-RISC builds upon the assumption that resilience is a personal quality that reflects one’s ability or capacity to successfully cope with stress and adversity (Connor and Davidson 2003). Drawing mainly on the seminal work of Suzanne Kobasa, Michael Rutter, and David Lyons, the CD-RISC captures qualities such as hardiness (e.g., commitment, viewing change as challenge, control), self-efficacy, goal and action orientation, tolerance of negative affect, patience, sense of humor in the face of stress, and tendency to bounce back from stress (Connor and Davidson 2003). Subsequent research established the CD-RISC’s good reliability, convergent, divergent, and criterion validity, and sensitivity to change in response to treatment (Davidson 2018). However, the suggested five-factor structure of the CD-RISC (i.e., personal competence, trust/affect tolerance/strengthening effects of stress, acceptance of change and secure relationships, control, and meaning) turned out to be unstable across samples (e.g., Campbell-Sills and Stein 2007; Davidson 2018).

In response to the unstable factor structure, Campbell-Sills and Stein (2007) developed a refined brief version of the CD-RISC: The CD-RISC-10 is focused on persistence and hardiness and the retained 10 items reflect tolerance of negative experiences such as failure, pressure, change, personal problems, and painful feelings. These qualities are compatible with an ability or capacity to bounce back from stress. Prior research established the good to excellent reliability, convergent, divergent, and criterion validity, and, with few exceptions, the unifactorial structure of the CD-RISC-10 (e.g., Campbell-Sills and Stein 2007; Davidson 2018; Hébert et al. 2018; Kuiper et al. 2019; Madewell and Ponce-Garcia 2016; Sarubin et al. 2015; Ye et al. 2017). Moreover, the total scores of the CD-RISC and CD-RISC-10 correlate usually about .90 (e.g., Kuiper et al. 2019; Madewell and Ponce-Garcia 2016), suggesting that the CD-RISC-10 provides a good proxy for the CD-RISC total score.

Parallel to the CD-RISC-10, Vaishnavi et al. (2007) introduced the ultra-brief CD-RISC-2. The two selected items are supposed to reflect the essence of resilience (i.e., able to bounce back after stress and to adapt to change). Although items were chosen on subjective and not on empirical grounds, the CD-RISC-2 shows moderate levels of reliability, validity, and agreement with the CD-RISC (e.g., Davidson 2018; Kuiper et al. 2019; Vaishnavi et al. 2007). Compared to the CD-RISC-10, the CD-RISC-2 yields a less broad coverage of trait resilience, poorer psychometric properties, and lower agreement with the CD-RISC, which led Kuiper et al. (2019) to conclude that research may focus on the CD-RISC-10 and that the CD-RISC-2 is preferable for situations where completion time is critical.

In their methodological review, Windle et al. (2011) ranked the CD-RISC and CD-RISC-10 among the top three resilience scales with the best psychometric quality. Due to their high psychometric quality, the CD-RISC and especially the CD-RISC-10 are probably the most widely used resilience scales (Salisu and Hashim 2017). To date, the CD-RISC has been translated into over 70 languages (Davidson 2018). Using a sample of *N* = 201 healthy adults, Sarubin et al. (2015) provide evidence for the reliability (Cronbach’s α, α = .84, and test-retest-reliability, *r*_tt_ = .81) and validity of the German CD-RISC-10 (data for the CD-RISC were also reported, the CD-RISC-2 was omitted). However, they relied on principal component analysis to test for factorial validity and they drew only on the Big-Five trait factors and the Resilience Scale 25 (RS-25; Wagnild and Young 1993) to establish convergent and discriminant validity. Given that the German CD-RISC-10 is in active use (e.g., Henninger and Plieninger in press; Matzka et al. 2016; Peter et al. 2018), a more thorough examination of its validity is highly needed. Harrer et al. (2018) used the German CD-RISC-2, but aside from descriptive statistics and change in response to a stress management intervention, no further psychometric details were provided. Given this additional lack of evidence for the psychometric quality of the CD-RISC-2, the present study aims to add to the psychometric literature on the German CD-RISC-10 and CD-RISC-2 by drawing on a larger sample, confirmatory factor analysis, more varied criteria, tests of incremental validity, and tests of the buffer effect of trait resilience.

### Hypotheses

Prior evidence suggests a unifactorial structure of the CD-RISC-10 (e.g., Campbell-Sills and Stein 2007; Davidson 2018; Hébert et al. 2018; Madewell and Ponce-Garcia 2016; Ye et al. 2017). We therefore expect that a unifactorial model of the German CD-RISC-10 will fit adequately to the data (***H1***).

The RS-25 (Wagnild and Young 1993) assesses trait resilience as reflected by someone’s personal competence and acceptance of self and life, which promote an individual’s coping capacity and adaptation to adversity. The RS-25 shows good psychometric quality (Windle et al. 2011), and strong convergence with the CD-RISC-10 (i.e., *r* ≥ .60; Madewell and Ponce-Garcia 2016; Sarubin et al. 2015). Von Eisenhart Rothe et al. (2013) introduced a brief version of the RS-25 that consists of five items and captures the two originally postulated RS-25 factors by at least one item. We expect moderate to strong positive correlations between the RS-5 and the CD-RISC-10 (***H2***) which would indicate convergent validity.

Trait emotional intelligence (*TEI*) refers to a distinct, compound construct that lies at lower levels of trait hierarchies and covers people’s dispositions or trait self-efficacies of how they experience and utilize affect-laden information (Petrides et al. 2007). The TEI sampling domain comprises 15 facets, of which 13 fall under four factors (Petrides 2009): *self-control* (emotion regulation, stress management, low impulsiveness), *well-being* (self-esteem, trait happiness, optimism), *sociability* (emotion management, social awareness, assertiveness), and *emotionality* (emotion perception, emotion expression, trait empathy, relationships). The four TEI factors along with the facets of self-motivation and adaptability are located under a global TEI factor (Jacobs et al. 2015; Petrides 2009). Trait resilience overlaps conceptually with several TEI facets, in particular with emotion regulation, low impulsiveness, stress management, self-esteem, optimism, assertiveness, adaptability, and self-motivation (cf., Connor and Davidson 2003). This implies convergent relations between trait resilience and global TEI (e.g., Di Fabio and Saklofske 2014), self-control, well-being, and sociability. Emotionality seems to be of limited relevance and thus divergent to trait resilience. We thus expect moderate to strong correlations between CD-RISC-10 with global TEI, self-control, well-being, and sociability, and small positive correlations with emotionality (***H3***).

Some authors discuss good mental health as a proxy for resilience (Aburn et al. 2016). This view is consistent with meta-analytical results that trait resilience has moderate to strong negative relations with perceived stress and negative mental health indicators (e.g., anxiety, depression, negative affect) and moderate to strong positive relations with positive mental health indicators (e.g., life satisfaction, positive affect) (e.g., Hu et al. 2015; Lee et al. 2013). We thus expect that the CD-RISC-10 shows moderate to strong negative correlations with perceived stress, depression, generalized anxiety, and sleep problems, and moderate to strong positive associations with life satisfaction, well-being, and mental health related quality of life (mental *HRQoL*; ***H4a***), signaling convergent validity. Psychological resilience is related to, but differs from physical resilience (i.e., the ability of the body to build, maintain, and repair itself and to recover from illness or injury or to maintain physical health in the face of adversity; e.g., Resnick et al., 2011). Hence, trait resilience has only small to moderate effects on physical health (e.g., Velickovic et al. 2020). We thus expect weak to moderate positive relations between CD-RISC-10 and physical HRQoL (***H4b***), signaling discriminant validity.

The RS-5 and global TEI are important predictors of various health outcomes (e.g., Martins et al. 2010; von Eisenhart Rothe et al. 2013). However, we expect that associations between the CD-RISC-10 and health indicators will remain significant even if we control for overlap with age, sex, and the RS-5 and even with global TEI (***H5***). Finding significant partial effects would provide support for the incremental validity of the German CD-RISC-10.

Resilience is supposed to act as a buffer that protects individuals’ mental health following exposure to adversity (e.g., Aburn et al. 2016). Associations between exposure to adversity and mental health problems are thus supposed to be stronger at lower levels of trait resilience than at higher levels of trait resilience. This buffer hypothesis has been confirmed in a recent meta-analysis (Hu et al. 2015) and in several subsequent studies (e.g., Hébert et al. 2018; Li et al. 2019). We thus expect that effects of perceived stress on life satisfaction, mental health problems, and physical HRQoL will be stronger at lower than at higher levels of trait resilience (***H6***), which would provide further support for the validity of the German CD-RISC-10.

The CD-RISC-2 covers key aspects of resilience (Vaishnavi et al. 2007) and it shows acceptable psychometric properties (e.g., Davidson 2018). But given the lower reliability of the CD-RISC-2, we expect that its associations with the selected criteria follow the pattern obtained for the CD-RISC-10, but with attenuated effect sizes (***H7***).

## Methods

### Participants and Procedure

Data were collected from February to June 2016 at two universities in Germany by the means of convenience sampling and by snow-ball sampling in students’ own networks of acquaintances. Subjects were invited to participate if they were currently enrolled as students at a university or a university of applied sciences and speak German fluently. Only individuals who provided informed consent were granted access to the survey. Subjects completed a web-based questionnaire containing several scales on personality and health. Completion of the survey took, on average, 30 min. The order of the scales was randomized in order to control for participant response fatigue. No material incentive was given for participation. Through the online platform EFS Questback, a total of 381 valid and complete questionnaires were received. However, 21 subjects were not enrolled as a student and were thus excluded, leading to a final sample of *N* = 360 students (259 females and 101 males). Participants were *M* = 22.34 years old (range: 18 to 41 years; *SD* = 2.81). The majority indicated that they were students of psychology (*n* = 221), followed by economics (*n* = 44), and law (*n* = 14).

### Measures

#### Connor-Davidson Resilience Scale-10 (CD-RISC-10; Campbell-Sills and Stein 2007)

The CD-RISC-10 is a brief version of the CD-RISC (Connor and Davidson 2003). It consists of 10 items reflecting the ability to tolerate experiences such as painful feelings, pressure, illness, change, or failure (German items are shown in Sarubin et al. 2015, p. 117). High scores are supposed to reflect an ability to bounce back from stress and adversity. Participants indicated on a 5-point Likert-type scale (0=‘not true at all’ to 4=‘true nearly all the time’) the extent to which each statement applies to them in general. We also created the *CD-RISC-2* score (Vaishnavi et al. 2007), consisting of item 1 (able to adapt to change) and item 5 (tend to bounce back after illness or hardship), which capture the hardiness aspect of resilience.

#### Resilience Scale-5 (RS-5; von Eisenhart Rothe et al. 2013)

The RS-5 is a brief version of the Resilience Scale (Wagnild and Young 1993). It measures trait resilience with five items (e.g., ‘Keeping interested in things is important to me.’). Subjects indicated on a 7-point Likert-type scale (1 = ‘strongly disagree’ to 7 = ‘strongly agree’), how much each statement applies to them. Prior research established the reliability and validity of the RS-5 (e.g., von Eisenhart Rothe et al. 2013). In the present sample, the reliability of the RS-5 was acceptable (see Table 1 for descriptive statistics and reliability of study variables).

**Table 1:** Descriptive statistics and reliability of study variables

	*M*	*SD*	α	ϖ
Trait resilience (CD-RISC-10)	2.76	0.54	.81	.82
Trait resilience (CD-RISC-2)	3.07	0.63	.48	.49
Trait resilience (RS-5)	5.71	0.82	.70	.71
Trait emotional intelligence (TEIQue-SF)	5.32	0.64	.87	.88
Well-being	5.65	0.94	.84	.85
Self-control	4.91	0.85	.58	.61
Emotionality	5.44	0.85	.70	.73
Sociability	5.22	0.82	.63	.64
Perceived stress (PSS-4)	1.56	0.71	.73	.74
Sleep problems (JSQ)	1.65	1.00	.71	.74
Depression (PHQ-9)	0.75	0.48	.82	.83
Generalized anxiety (GAD-7)	0.70	0.57	.86	.86
Life satisfaction (L-1 single item)	8.43	2.00	n.a.	n.a.
Well-being (WHO-5)	2.86	0.93	.83	.84
Physical HRQoL (SF-8)^a^	52.15	7.26	n.a.	n.a.
Mental HRQoL (SF-8)^a^	46.04	10.95	n.a.	n.a.

*Notes:* CD-RISC = Connor-Davidson Resilience Scale, GAD-7 = Generalized Anxiety Disorder Scale-7, HRQoL = Health related quality of life, JSQ = Jenkins Sleep Questionnaire, PHQ-9 = Patient Health Questionnaire-9, PSS-4 = Perceived Stress Scale-4, RS-5 = Resilience Scale-5, SF-8 = Short Form-8 Health Survey, TEIQue-SF = Trait Emotional Intelligence Questionnaire-Short Form, WHO-5 = World Health Organization Well-Being Index. ^a^ Both SF-8 component summary scores were calculated as linear combinations using weighted item scores and the algorithmic norm-based scoring method (Ware et al. 2001), estimates α and ϖ are thus not available

#### Trait Emotional Intelligence Questionnaire-Short Form (TEIQue-SF; Petrides 2009)

The TEIQue-SF provides a concise assessment of global TEI, and a rough assessment of the TEI factors of self-control, emotionality, well-being, and sociability. Subjects scored each of the 30 items (e.g., ‘Expressing my emotions with words is not a problem for me.’) on a 7-point Likert-type scale (1 = ‘absolutely disagree’ to 7 = ‘absolutely agree’). Reliability and validity of the German TEIQue-SF have been shown in previous research (e.g., Jacobs et al. 2015). In the present data, reliability of global TEI and the TEI factors was marginal to good.

#### Perceived Stress Scale-4 (PSS-4; Cohen et al. 1983; German PSS-4: Engling 2010)

Perceived stress was measured with four items, using a 12-month recall format (e.g., ‘In the last 12 months, how often have you felt that you were unable to control the important things in your life?’) and a five-point frequency scale (0 = ‘never’ to 4 = ‘very often’). Acceptable psychometric properties of the PSS-4 have been demonstrated (e.g., Demkowicz et al. in press). In the present survey, the reliability of the PSS-4 was acceptable.

#### Jenkins Sleep Questionnaire (JSQ; Jenkins et al. 1988)

The JSQ was administered to assess the presence of sleep disturbances during the last month (e.g., problems falling asleep, problems staying asleep). Each of the four items was scored on a 6-point frequency scale (0=‘not at all’ to 5 = ‘22–31 days’). Reliability and validity of the German JSQ are summarized in Becker et al. (2014). In the present study, the JSQ showed satisfactory reliability.

#### Patient Health Questionnaire-9 (PHQ-9; Kroenke et al. 2001)

The PHQ-9 assesses the presence of major depression. Subjects indicated for each of the nine items (e.g., depressed mood, anhedonia, feeling of tiredness) whether the depressive symptom has bothered them during the last two weeks (0=‘not at all’ to 3=‘nearly every day’). Good psychometric properties of the German PHQ-9 have been documented in prior research (e.g., Kocalevent et al. 2013), and reliability in the present study was good.

#### World Health Organization Well-Being Index (WHO-5; World Health Organization 1998)

The WHO-5 is a generic measure of well-being, and it can be used as a screening tool for depression (Brähler et al. 2007). Using a 6-point Likert-type scale (0=‘at no time’ to 5=‘at all time’), participants rated the five statements on how they had felt over the past two weeks (e.g., ‘… calm and relaxed’). The German WHO-5 has demonstrated good psychometric properties (e.g., Brähler et al. 2007), and its reliability in the present sample was good.

#### *Generalized Anxiety Disorder Scale-7 (GAD-7;* Spitzer et al. 2006*)*

The GAD-7 was administered to assess the presence of generalized anxiety disorder symptoms. Subjects rated for each of the seven items (e.g., worrying too much about different things) whether the described symptom has bothered them during the previous two weeks (0=‘not at all’ to 3=‘nearly every day’). Evidence for the validity and reliability of the German GAD-7 has been provided by Löwe et al. (2008). In the present data, reliability of the GAD-7 was good.

#### Life Satisfaction Short Scale (L-1; Beierlein et al. 2014)

General satisfaction with life was measured by a single item (‘How satisfied are you at present, all in all, with your life?’), which was rated on an 11-point scale (1=‘not satisfied at all’ to 11 = ‘completely satisfied’). Evidence for the validity and for acceptable levels of test-retest reliability (r_tt_ = .67) of the L-1 has been reported in Beierlein et al. (2014).

#### Short Form-8 Health Survey (SF-8; Ware et al. 2001)

The SF-8 was administered to assess mental and physical HRQoL. Each single-item assesses one of the eight dimensions of the original SF-36 health survey (e.g., bodily pain, vitality, mental health). Items were scored on a 5- or 6-point scale (4-week recall format). Physical (PCS) and mental (MCS) component summary scores were derived by using an algorithmic norm-based scoring method (Ware et al. 2001) and higher scores indicate better HRQoL. Prior research showed the reliability and validity of the German SF-8 (e.g., Ellert et al. 2005). Reliability for the overall SF-8, assessed by Cronbach’s alpha and coefficient omega, was 0.84 and 0.85, respectively.

### Statistical Analysis

First, item characteristics of the CD-RISC-10 were examined. Second, Cronbach’s α and McDonald’s ω of the CD-RISC-10 and CD-RICS-2 were calculated using JASP version 0.10. Third, factorial validity of the CD-RISC-10 was shown by the means of confirmatory factor analysis (CFA), performed on the variance-covariance matrix using Mplus 8 (Muthén and Muthén 1998-2017). To evaluate model fit, the robust Satorra-Bentler scaled χ^2^-statistic was complemented by three fit indices: root mean square error of approximation (RMSEA), standardized root mean square residual (SRMR), and comparative fit index (CFI). A well-fitting model is indicated by RMSEA≤.06, SRMR≤.08, and CFI ≥ .95 (e.g., Brown 2006). Factorial invariance with the original CD-RISC-10 was quantified with Tucker’s phi using the Invariance app (Watkins 2005). Finally, using FACTOR 10 (Ferrando and Lorenzo-Seva 2017) we backed the adequacy of the unifactorial solution by three different approaches: a) parallel analysis based on 1000 random correlation matrices obtained from permutation of raw data, minimum rank factor analysis, and a 95%-threshold (Timmerman and Lorenzo-Seva 2011), b) Schwarz’s Bayesian Information Criterion (BIC) dimensionality test (i.e., the model with the smallest BIC was chosen), and c) the Hull method (based on robust CFI), which aims to identify the number of common factors which optimizes the balance between model fit and number of parameters (Lorenzo-Seva et al. 2011).

Fourth, convergent and divergent validity was established with Pearson correlations between both brief CD-RISC versions and criterion variables using IBM-SPSS version 22. Fifth, incremental validity was shown by partial correlations between both brief CD-RISC scores and criterion variables, controlling for age, sex, RS-5, and global TEI. Finally, the hypothesis that trait resilience buffers the effect of perceived stress on life satisfaction, and mental and physical health was tested in a series of multiple regression analyses. Each regression analysis included age, sex, the mean-centered PSS-4 and brief CD-RISC scores, and the PSS-4 x CD-RISC interaction term as predictors, and was carried out in PROCESS version 3, using robust standard errors (HC4; Hayes 2018). A significant interaction term indicates the presence of a buffer effect and simple slopes at different levels of resilience were used to interpret the buffer effect. Prior to the regression analyses, all mental health scale scores were submitted to a scale-level principal component analysis (PCA), component scores were saved via regression, and used in the subsequent regression analysis as aggregated mental health outcome. In all analyses, an a priori significance level of α = .05 was chosen.

## Results

### Item Characteristics, Reliability, and Confirmatory Factor Analysis

Characteristics of the CD-RISC-10 items are shown in Table 2. Item means (difficulty indicators) varied between 2.31 and 3.20 and all exceeded the scale midpoint (2 = *sometimes true*). Except for item 5, skew and kurtosis at item level were low, leading to negligible skew and kurtosis in the CD-RISC-10 score (skew = −0.20, and kurtosis = 0.004). Inter-item correlations ranged from *r* = .15 to *r* = .48 (mean *r* = .30). All corrected item-total-correlations (discrimination parameters) exceeded the critical value of *r*_it_ = .30 (range *r*_it_: .38 to .60). The reliability of the CD-RISC-10 was good, α = .81 and ω = .82.

**Table 2:** Psychometric properties of the CD-RISC-10 items

CD-RISC-10 item description^a^	*M*	*SD*	Skew	Kurt	*r*_it_	λ	*R*^2^
1. Able to adapt to change	4.13	0.70	−0.47	0.17	.46	.51	.26
2. Can deal with whatever comes	3.76	0.81	−0.51	0.25	.60	.68	.46
3. Tries to see humorous side of problems	3.46	0.98	−0.01	−0.73	.40	.44	.19
4. Coping with stress can strengthen me	3.50	0.97	−0.24	−0.25	.40	.45	.20
5. Tend to bounce back after illness or hardship	4.01	0.85	−0.92	1.08	.38	.44	.19
6. Can achieve goals despite obstacles	4.20	0.71	−0.63	0.27	.54	.60	.36
7. Can stay focused under pressure	3.60	0.94	−0.45	−0.10	.48	.54	.29
8. Not easily discouraged by failure	3.31	0.99	−0.10	−0.57	.51	.58	.33
9. Thinks of self as strong person	3.91	0.91	−0.66	0.06	.60	.69	.47
10. Can handle unpleasant feelings	3.73	0.95	−0.33	−0.51	.54	.59	.35

*Notes*: CD-RISC = Connor-Davidson Resilience Scale. *M =* Mean, *SD =* standard deviation; Kurt = excess kurtosis; *r*_it_ = corrected item-total correlation; λ *=* standardized CFA factor loadings (unifactorial model)*; R*^2^ = item variance explained by the resilience factor. ^a^ Items were taken from the German CD-RISC (Sarubin et al. 2015, p. 117), item descriptions were taken from Campbell-Sills and Stein (2007, p. 1025)

Parallel analysis, the BIC dimensionality test, and the Hull method consistently suggested one factor to retain. We will therefore focus at the unidimensional model of the CD-RISC-10 and dismiss more complex factorial models. The unifactorial model missed perfect model fit, robust χ^2^(35) = 73.56, *p* < .001, but approximate fit was good in terms of RMSEA = .055 and SRMR = .042, and fair in terms of CFI = .944. The resilience factor accounted for 19% to 47% of variance in the CD-RISC-10 items and factor loadings ranged between 0.44 and 0.69 (see Table 2). Tucker’s phi suggests that the factor loadings and the factor loadings of the original CD-RISC-10 (Campbell-Sills and Stein 2007, p. 1025) can be considered as equal, φ = 0.99 (Lorenzo-Seva and Ten Berge 2006).

Item 1 and item 5, that form the CD-RISC-2, were moderately related, *r* = .32, *p* < .001, and showed fair loadings on the resilience-factor. The reliability of the CD-RISC-2 thus fell below the critical threshold of .50 (Table 2). The agreement between the CD-RISC-10 and CD-RISC-2 was moderate, *r* = .65, *p* < .001.

### Convergent, Divergent, and Incremental Validity

Older subjects and males indicated higher CD-RISC-10 scores (for effects see Table 3). The correlation between CD-RISC-10 and RS-5 was *r* = .44, *p* < .001, indicating moderate convergence. The CD-RISC-10 showed a strong association with global TEI, *r* = .63, *p* < .001, and weak to strong correlations with all TEI factors (*r* = .24 to .59, all *p*s < .001). The CD-RISC-10 score correlated positively with positive indicators of mental health (i.e., WHO-5, L-1, MCS) and PCS, and negatively with perceived stress and negative indicators of mental health (i.e., PHQ-9, GAD-7, and JSQ). These correlations were moderate to large in size implying convergent validity, except for emotionality, sleep problems, and physical HRQoL, where small effect sizes suggested divergent validity.

**Table 3:** Correlations and partial correlations of the CD-RISC-10 and CD-RISC-2 with study variables

	CD-RISC-10			CD-RISC-2		
	*r*	*pr*_1_	*pr*_2_	*r*	*pr*_1_	*pr*_2_
Age	.12^*^	n.a.	n.a.	.03	n.a.	n.a.
Sex (males = 0, females = 1)	−.17^***^	n.a.	n.a.	−.02	n.a.	n.a.
Trait resilience (RS-5)	.44^***^	n.a.	n.a.	.32^***^	n.a.	n.a.
Trait emotional intelligence (TEIQue-SF)	.63^***^	.55^***^	n.a.	.51^***^	.43^***^	n.a.
Well-being	.57^***^	.48^***^	n.a.	.47^***^	.38^***^	n.a.
Self-control	.59^***^	.50^***^	n.a.	.40^***^	.32^***^	n.a.
Emotionality	.24^***^	.17^**^	n.a.	.29^***^	.22^***^	n.a.
Sociability	.46^***^	.32^***^	n.a.	.34^***^	.24^***^	n.a.
Perceived stress (PSS-4)	−.49^***^	−.41^***^	−.12^*^	−.39^***^	−.32^***^	−.14^**^
Sleep problems (JSQ)	−.24^***^	−.12^*^	−.18^***^	−.21^***^	−.14^**^	−.05
Depression (PHQ-9)	−.42^***^	−.30^***^	−.22^***^	−.39^***^	−.31^***^	−.18^**^
Generalized anxiety (GAD-7)	−.39^***^	−.33^***^	−.08	−.36^***^	−.31^***^	−.16^**^
Life satisfaction (L-1 single item)	.41^***^	.33^***^	.20^**^	.30^***^	.22^***^	.03
Emotional well-being (WHO-5)	.48^***^	.38^***^	.28^***^	.39^***^	.29^***^	.14^**^
Physical HRQoL (SF-8)	.12^*^	.10	.01	.19^***^	.18^***^	.15^**^
Mental HRQoL (SF-8)	.46^***^	.38^***^	.13^*^	.32^***^	.25^***^	.09

*Notes*: CD-RISC = Connor-Davidson Resilience Scale, GAD-7 = Generalized Anxiety Disorder Scale-7, HRQoL = Health related quality of life, JSQ = Jenkins Sleep Questionnaire, PHQ-9 = Patient Health Questionnaire-9, PSS-4 = Perceived Stress Scale-4, RS-5 = Resilience Scale-5, SF-8 = Short Form-8 Health Survey, TEIQue-SF = Trait Emotional Intelligence Questionnaire-Short Form, WHO-5 = World Health Organization Well-Being Index. *pr*_1_ = partial correlations controlled for Resilience Scale-5, sex, and age; *pr*_2_ = partial correlations controlled for global TEI, Resilience Scale-5, sex, and age. n.a. = not available

^*^p < .05; ^**^p < .01; ^***^p < .001 (2-tailed)

Except for age and sex, the CD-RISC-2 also correlated significantly with all study variables (Table 3). Using the *z*-test for dependent correlations, the mean absolute correlation involving the CD-RISC-2, mean |*r*| = .31, was significantly smaller than its CD-RISC-10 counterpart, mean |*r*| = .40, *z* = −2.22, *p* = .026 (when only correlations with perceived stress and health indicators were included, both mean correlations no longer differed, mean |*r*| = .32 vs. mean |*r*| = .38, *z* = −1.50, *p* = .134). However, after correcting for unreliability of both scale scores, both mean absolute disattenuated correlations were comparable (.43 vs. .44), suggesting that the lower validity of the CD-RISC-2 was due to its poorer signal-to-noise ratio.

To test for incremental validity, partial correlations between CD-RISC-10 and criterion variables were considered. When age, sex, and RS-5 were controlled (see *pr*_1_ in Table 3), all but one partial correlation remained statistically significant, providing support for the incremental validity of the CD-RISC-10. The only exception was found for physical HRQoL, which just missed significance, *pr*_1_ = .10, *p* = .07. When global TEI was additionally controlled for, six out of eight partial correlations remained significant, suggesting incremental validity of the CD-RISC-10 even beyond global TEI, RS-5, age, and sex (see *pr*_2_ in Table 3).

Concerning the CD-RISC-2, all partial correlations controlling for age, sex, and RS-5 (see *pr*_1_ in Table 3), and five partial correlations controlling for g, sex, RS-5, and global TEI reached significance (see *pr*_2_ in Table 3). However, the mean absolute partial correlation tended to be lower for CD-RISC-2 than for CD-RISC-10 (mean |*pr*_1_|: .28 vs. .34; mean |*pr*_2_|: .12 vs. .15), suggesting slightly lower incremental validity of the CD-RISC-2.

### Buffer Effect of Trait Resilience

To form an aggregated mental health indicator, the relevant scale scores (i.e., WHO-5, JSQ, PHQ-9, GAD-7, and MCS) were submitted to a scale-level PCA. The Kaiser-Meyer-Olkin measure of sampling adequacy indicated the data as suitable for PCA, KMO = .85. One component with an eigenvalue >1.00 was retained (eigenvalue = 3.39), which explained 67.86% of the variance. This component was labeled *mental health problems*, defined by positive loadings of the PHQ-9 (0.90), GAD-7 (0.84), and JSQ (0.70) scores and negative loadings of the MCS (−0.84) and WHO-5 (−0.82) scores. The component scores were saved via regression and used in the subsequent regression analyses.

**Fig. 1:**
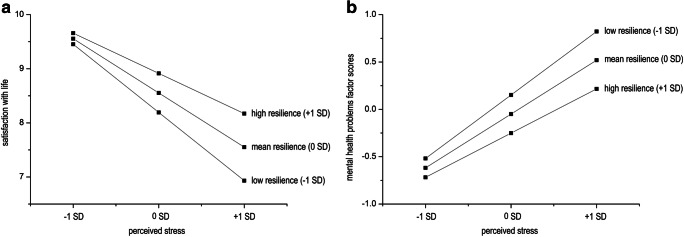
Effects of perceived stress on satisfaction with life (plot a) and mental health problems component scores (plot b), depending on levels of trait resilience

To test the buffer effect of trait resilience, three regression analyses were conducted. Results are presented in Table 4. Perceived stress had a negative and trait resilience a positive effect on life satisfaction, qualified by a significant stress x trait resilience interaction. Simple slope analysis indicated that the negative effect of perceived stress on life satisfaction was stronger at lower levels of trait resilience (−1 *SD*), *b* = −1.79, *p* < .001, than at higher levels of trait resilience (+1 *SD*), *b* = −1.04, *p* < .001 (see Fig. 1, plot a). For mental health problems, perceived stress had a positive and trait resilience a negative effect, qualified by a significant stress x trait resilience interaction. Simple slope analysis showed that perceived stress had a positive effect on mental health problems, and this effect was stronger at lower levels of trait resilience, *b* = 0.95, *p* < .001, than at higher levels of trait resilience, *b* = 0.66, *p* < .001 (see Fig. 1, plot b). For physical HRQoL, none of the predictor variables were significant.

**Table 4:** Regressing life satisfaction, mental health problems, and physical HRQoL on age, sex, perceived stress, trait resilience (CD-RISC-10), and trait resilience x perceived stress interaction

	Life satisfaction					Mental health problems					Physical HRQoL (SF-8)				
	*Β*	*SE*	95% CI	*r*	*sr*^2^	*Β*	*SE*	95% CI	*r*	*sr*^2^	*Β*	*SE*	95% *CI*	*r*	*sr*^2^
Age in years	−0.06^*^	0.03	[−0.11, −0.01]	−.02	.01	0.00	0.01	[−0.02, 0.03]	−.07	.00	−0.09	0.13	[−.35, 0.17]	−.01	.00
Sex (females)	0.46^*^	0.20	[0.07, 0.85]	.04	.01	−0.03	0.08	[−0.19, 0.13]	.07	.00	−1.38	0.79	[−2.93, 0.18]	−.10	.01
Perceived Stress	−1.41^***^	0.18	[−1.76, −1.06]	−.59^***^	.19	0.80^***^	0.07	[0.66, 0.94]	.67^***^	.25	−0.78	0.68	[−2.11, 0.55]	−.11^*^	.00
Resilience	0.67^***^	0.20	[0.28, 1.07]	.41^***^	.02	−0.38^***^	0.09	[−0.56, −0.20]	−.49^***^	.03	1.11	0.89	[−0.64, 2.86]	.12^*^	.00
Resilience x stress	0.68^***^	0.19	[0.30, 1.06]	.20^***^	.02	−0.27^***^	0.07	[−0.41, −0.12]	−.18^***^	.01	−1.26	0.71	[−2.67, 0.14]	−.06	.01
*R*^2^	0.40					0.50					0.03				
*F*(5, 354)	39.03^***^					79.90^***^					2.88^*^				

*Notes*. Life satisfaction = L-1 single item, Mental health = mental health component score, HRQoL = Health related quality of life, SF-8 = Short Form-8 Health Survey, *B* = unstandardized regression coefficient, *SE* = heteroscedasticity consistent HC4 standard error, 95% CI = limits of the 95% confidence interval, *r* = Pearson correlation, *sr*^2^ = utility (squared semi-partial correlation), *R*^2^ = squared multiple correlation. ^*^p < .05, ^***^p < .001

When the analyses were carried out with the CD-RISC-2, the stress x trait resilience interaction was just significant for mental health problems, *b* = −0.15, *t*(354) = −1.98, *p* = .049, just failed significance for life satisfaction, *b* = 0.46, *t*(354) = 1.94, *p* = .053, and in line with the analysis involving the CD-RISC-10, missed significance for physical HRQoL, *b* = −0.43, *t*(354) = −0.43, *p* = .66. The effect of stress on mental health problems was stronger at low levels of trait resilience, *b* = 0.93, *p* < .001, than at high levels of trait resilience, *b* = 0.78, *p* < .001.

## Discussion

The CD-RISC and its authorized 10-item and 2-item versions are frequently used and psychometrically sound instruments to measure trait resilience (Davidson 2018). The present study aimed to underpin the psychometric properties of both brief German CD-RISC versions, as evidence for their psychometric properties is either limited (CD-RISC-10; e.g., Sarubin et al. 2015) or lacking (CD-RISC-2; e.g., Harrer et al. 2018), which hampers their use in medical and psychological research.

### The Psychometric Properties of the German CD-RISC-10

Consistent with the literature (e.g., Salisu and Hashim 2017; Windle et al. 2011), the German CD-RISC-10 showed good to excellent psychometric properties: At item level, discriminatory parameters were reasonably high and item difficulties were in the medium range. Reliability in terms of Cronbach’s alpha and McDonald’s omega were above .80 and can thus be regarded as good. In the CFA, the unifactorial structure of the CD-RISC-10 fitted acceptably to the data (***H1*** supported). Given that Sarubin et al. (2015) draw on a relatively small sample and used PCA to establish the unifactorial structure, the present CFA results provide important additional support for the factorial validity of the German CD-RISC-10.

However, although all items seem to measure a unitary construct, the less than perfect model fit points at the existence of small amounts of systematic error variance, which may have reduced the overall fit of the unifactorial model (e.g., Ye et al. 2017).

Convergent and divergent validity of the CD-RISC-10 was established by correlations with various clinical and non-clinical constructs that extend Sarubin et al.’s (2015) attempts to validate the German CD-RISC-10. The positive association with the RS-5 was moderate in size (***H2*** supported), but fell below the large effect sizes that are usually found with brief versions of the Resilience Scale (e.g., Madewell and Ponce-Garcia 2016). This is likely due to the reduced content validity of the RS-5: Item selection was mainly based on optimizing Cronbach’s α and led to the retention of two items with almost identical content referring to interest (von Eisenhart Rothe et al. 2013), which narrows the RS-5’s breadth of coverage. The strong association between the CD-RISC-10 and the TEIQue-SF matched the respective correlation of *r* = .67 found in an Italian sample using the same instrumentation (Di Fabio and Saklofske 2014). When TEI factors were considered, the strongest correlations with the CD-RISC-10 emerged with self-control and well-being, followed by sociability and emotionality (***H3*** supported). The results mirror the close nexus of the former with the content domain of trait resilience (cf. Connor and Davidson 2003), whereas emotionality seems to be of limited relevance for trait resilience. This result is consistent with the recent finding that the bulk of global TEI’s effects on various functioning-related criteria are mainly due to self-control and well-being (Andrei et al. 2016), which also showed the strongest overlap with trait resilience.

The CD-RISC-10 evidenced moderate to strong negative associations with perceived stress, depression, and generalized anxiety, moderate to strong positive associations with life satisfaction, well-being, and mental HRQoL, and a small positive association with physical HRQoL. These associations are consistent with prior evidence (e.g., Hu et al. 2015; Lee et al. 2013) and they provide support for the convergent and discriminant validity of the German CD-RISC-10 (***H4a*** and ***H4b*** supported). The only unexpected result was the weak association of trait resilience with sleep quality, which is usually moderate to strong in size (e.g., Li et al. 2019). It is currently unclear whether this deviation reflects idiosyncrasies of the sample or a method effect (i.e., brief scale with limited content coverage). Except for physical HRQoL, all associations remained significant beyond the overlap with age, sex, and RS-5. When overlap with global TEI was additionally partialed out, only the associations with physical HRQoL and generalized anxiety became non-significant. Given that the RS-5 and global TEI are themselves important predictors of health-related outcomes (e.g., von Eisenhart Rothe et al. 2013; Martins et al. 2010), the present results clearly help to establish the incremental validity of the German CD-RISC-10 (***H5*** partially supported).

Finally, trait resilience buffered the effect of perceived stress on life satisfaction and mental health problems, but not on physical HRQoL (***H6*** partially supported). Consistent with Hu et al. (2015), the significant buffer effects were also compatible with Fergus and Zimmerman’s (2005) protective-reactive model, where the protective factor diminishes, but not fully removes the association between a risk and an outcome. The results thus provide further evidence for the validity of the CD-RISC-10. The lacking buffer effect for physical HRQoL likely reflects the fact that psychological resilience and physical resilience are related, yet distinct constructs (Resnick et al., 2011), and that the former may bear limited relevance for physical health.

### The Psychometric Properties of the German CD-RISC-2

The level of agreement between the CD-RISC-10 and CD-RISC-2 was moderate and consistent to prior findings (e.g., Kuiper et al. 2019). The reliability of the CD-RISC-2 just missed the .50 threshold, unlike the original CD-RISC-2 (cf. Davidson 2018; Kuiper et al. 2019). But given that low internal consistency can be expected for an ultra-short scale that aims to retain the breadth of a multifaceted construct like trait resilience, test-retest reliability estimates of the German CD-RISC-2 may be more informative (Rammstedt and Beierlein 2014), and need to be shown in future research. The pattern of correlations and partial correlation with criterion variables was similar to the CD-RISC-10, albeit the absolute size of associations was, on average, smaller (***H7*** supported) and this overall difference was due to attenuation. Interestingly, the overall correlational difference was no longer significant when only health-related variables were considered, suggesting comparable validity of both brief CD-RISC versions despite their different reliability. This finding bolsters the notion that the CD-RISC-2 captures the essence of resilience (Vaishnavi et al. 2007). However, when the buffer hypothesis was tested, a just significant interaction term emerged only for mental health problems. Given that the reliability of a multiplicative interaction term is compromised when one or both of the constituent terms suffer from low reliability, the power to detect a buffer effect in the data was notably reduced when the CD-RISC-2 was used.

### Limitations, Implications, and Conclusions

This study has several limitations: First, the cross-sectional nature of the data prevents any causal claims. Especially the assumption that trait resilience buffers the effects of perceived stress on health outcomes needs to be shown in longitudinal studies. Second, we relied on self-reports, which may have introduced various method biases (e.g. Podsakoff et al. 2003). Future research might draw on varying methods and sources of information (e.g., informant ratings, standardized clinical interviews for clinical criteria). Third, data were collected online, which may have compromised the quality of the data. However, several studies show that online and paper and pencil surveys yield equivalent data (e.g., Weigold et al. 2013), which lends some credence to the present results. Fourth, the present study drew on a student sample and results may thus not generalize to older or clinical populations. Thus, measurement invariance of the CD-RISC-10 across different samples and across time as well as invariance of the associations between trait resilience and the selected criteria still need to be established. Finally, the current coronavirus-19 disease (COVID-19) pandemic seems to impact adversely on psychological resilience (e.g., Killgore et al. 2020; Kimhi et al. 2020). Therefore, when comparing CD-RISC-10 scale means, caution is advised and the time point of data collection should be taken into account (i.e., pre-pandemic vs. pandemic).The current coronavirus-19 disease (COVID-19) pandemic has a negative impact on many people’s mental health and subjective well-being (e.g., Hossain et al. 2020; Kimhi et al. 2020). However, people differ widely in how they respond to the perceived COVID-19 threat and its adverse consequences (e.g., Kimhi et al. 2020; Wang et al. 2020), which points at trait resilience as a key concept in this context: For example, trait resilience seems to relate negatively to mental health problems and worries about the adverse effects of COVID-19 (Killgore et al. 2020), and it seems to be negatively and specifically related to state anxiety during pandemic isolation beyond the effects of trait anxiety and experiential avoidance (Rotărescu et al. 2020). Consistent with the buffer effect of trait resilience, Paredes et al. (2021) showed that the negative indirect effect of perceived COVID-19 threats on subjective well-being through future anxiety was moderated by the level of trait resilience (i.e., the effect of perceived threats on future anxiety was stronger at lower levels of resilience). Finally, Kavčič et al. (2020) identified trait resilience as a crucial factor that promotes psychological functioning during COVID-19 pandemics. Building and stabilizing individual’s resilience to mitigate the adverse psychological impact of the COVID-19 pandemic is therefore an important endeavor and evidence-based recommendations for promoting resilience in the face of the COVID-19 pandemic are highly needed (e.g., Barthélemy et al. 2021; Killgore et al. 2020).

The CD-RISC and its short versions are currently vital for research on resilience, mental health, and resilience interventions under COVID-19 pandemic (e.g., Kavčič et al. 2020; Killgore et al. 2020; Kimhi et al. 2020; Paredes et al. 2021; Rotărescu et al. 2020). The present study adds to this line of research by expanding the psychometric literature on the German CD-RISC-10 and CD-RISC-2. It confirmed the good to excellent psychometric properties of the German CD-RISC-10. The CD-RISC-10 is thus recommended as a measure of trait resilience in a wide range of clinical and health psychology research contexts. However, the German CD-RISC-2 shows less desirable psychometric properties and it might be recommended only for situations when completion time is critical, when unreliability is accounted for in the measurement model, and when the buffer effect of trait resilience is not the focus of the study. Researchers are thus encouraged to critically consider whether the gain of about two minutes of completion time sufficiently compensates for the loss of psychometric rigor that the use of the CD-RISC-2 entails.

## Data Availability

Data and material that support the findings of this study are available from the corresponding author upon reasonable request.
